# Mathematical Modelling of Material Transfer to High-Density Lipoprotein (HDL) upon Triglyceride Lipolysis by Lipoprotein Lipase: Relevance to Cardioprotective Role of HDL

**DOI:** 10.3390/metabo12070623

**Published:** 2022-07-06

**Authors:** Svetlana Schekatolina, Viktoriia Lahovska, Aleksandr Bekshaev, Sergey Kontush, Wilfried Le Goff, Anatol Kontush

**Affiliations:** 1 Odessa National Technological University, 65000 Odessa, Ukraine; skontush@odessaglobe.com (S.S.); lagov.vika@gmail.com (V.L.); 2 Physics Research Institute, I.I. Mechnikov Odessa National University, 65082 Odessa, Ukraine; bekshaev@onu.edu.ua (A.B.); kontush@odessaglobe.com (S.K.); 3 Unité de Recherche sur les Maladies Cardiovasculaires, Institut National de la Santé et de la Recherche Médicale (INSERM), le Métabolisme et la Nutrition, ICAN, Sorbonne Université, F-75013 Paris, France; wilfried.le_goff@sorbonne-universite.fr

**Keywords:** mathematical modelling, lipolysis, triglyceride-rich lipoproteins, high-density lipoprotein, free cholesterol, lipoprotein lipase, triglycerides, intestine, atherosclerosis, cardiovascular disease

## Abstract

High-density lipoprotein (HDL) contributes to lipolysis of triglyceride-rich lipoprotein (TGRL) by lipoprotein lipase (LPL) via acquirement of surface lipids, including free cholesterol (FC), released upon lipolysis. According to the reverse remnant-cholesterol transport (RRT) hypothesis recently developed by us, acquirement of FC by HDL is reduced at both low and extremely high HDL concentrations, potentially underlying the U-shaped relationship between HDL-cholesterol and cardiovascular disease. Mechanisms underlying impaired FC transfer however remain indeterminate. We developed a mathematical model of material transfer to HDL upon TGRL lipolysis by LPL. Consistent with experimental observations, mathematical modelling showed that surface components of TGRL, including FC, were accumulated in HDL upon lipolysis. The modelling successfully reproduced major features of cholesterol accumulation in HDL observed experimentally, notably saturation of this process over time and appearance of a maximum as a function of HDL concentration. The calculations suggested that the both phenomena resulted from competitive fluxes of FC through the HDL pool, including primarily those driven by FC concentration gradient between TGRL and HDL on the one hand and mediated by lecithin-cholesterol acyltransferase (LCAT) and cholesteryl ester transfer protein (CETP) on the other hand. These findings provide novel opportunities to revisit our view of HDL in the framework of RRT.

## 1. Introduction

Lipoproteins are plurimolecular, quasi-spherical, pseudomicellar complexes composed of polar and non-polar lipids solubilised by apolipoproteins (apos) [1]. Lipoprotein metabolism in circulation contributes to all major aspects of human metabolism, which involve energy conversion together with anabolic and catabolic pathways. Material transfer across lipoproteins represents an important part of their metabolism; indeed, both lipid and protein molecules readily exchange between different lipoprotein classes under the action of enzymes and lipid transfer proteins, leading to alterations of lipoprotein composition, structure and biological function [2]. 

Major lipoproteins in human plasma are chylomicrons, very low-density lipoprotein (VLDL), low-density lipoprotein (LDL) and lipoprotein (a), which are commonly regarded as large, light, lipid-rich, apoB-containing particles, and high-density lipoprotein (HDL), a small, dense, protein-rich, apoA-I-containing particle [3]. Chylomicrons and VLDL are respectively produced by the intestine and the liver, LDL represents a product of VLDL catabolism, and HDL in part originates from the lipidation of apoA-I upon interaction with ATP-binding cassette transporters ABCA1 and ABCG1 present on cell membranes. Chylomicrons and VLDL are rich in triglycerides (TG) and are often referred to as TG-rich lipoprotein (TGRL); they may contain large amounts of apoA-I and other proteins typically associated with HDL [4,5]. Elevated plasma levels of apoB-containing lipoproteins and low levels of HDL-cholesterol (HDL-C) are widely accepted risk factors for cardiovascular disease [6,7,8,9]. However, recent epidemiological studies reveal that both cardiovascular and overall mortality is also increased at extremely high HDL-C levels, resulting in a non-linear U-shaped relationship with HDL-C [10,11,12]. This observation may account for negative results of HDL-C-raising trials which frequently feature extremely high on-treatment HDL-C concentrations [13,14].

Intravascular metabolism of lipoprotein classes is intervened via multiple pathways. In particular, metabolism of HDL is linked to that of TGRL via heteroexchange of core lipids mediated by cholesteryl ester transfer protein (CETP) [15] as well as via transfer to HDL of surface fragments of TGRL generated during lipolysis by lipoprotein lipase (LPL) [16,17,18]. Such interactions result in a negative correlation between circulating concentrations of HDL-C and TG [19]. The lipolytic pathway delivers to HDL high amounts of free cholesterol (FC) and phospholipid (PL), constituting a major source of HDL-C in humans [20,21]. Mechanistically, this activity can primarily be mediated by apoA-I, the major HDL protein and potent biological detergent possessing distinct lipid-binding properties [22,23,24].

The U-shaped relationship between HDL-C levels and cardiovascular risk can be explained in a framework of the reverse remnant-cholesterol transport (RRT) hypothesis recently developed by us [25,26]. According to this hypothesis, acquirement of surface TGRL remnants generated during lipolysis with subsequent transport of their FC to the liver constitutes a major biological activity of HDL which is impaired in subjects with both low and extremely high HDL-C levels. We postulated that this key metabolic pathway, which in part originates in the intestine with the secretion of apoA-I on chylomicrons, is essential for post- and interprandial TG metabolism and energy production [26]. The existence of an inverse U-shaped relationship between HDL concentrations and lipolytic FC transfer to HDL was demonstrated by us using an in vitro assay [25]. However, mechanisms underlying impaired FC transfer to HDL upon TGRL lipolysis by LPL remained poorly understood, despite pivotal biological and clinical roles of this pathway. 

Experimental studies of material transfer across lipoproteins are complicated by their rapid kinetics as well as by the requirement of lipoprotein separation before analysis. Mathematical models able to successfully simulate this process can therefore be of a special interest as they allow studying detailed kinetics of lipoprotein interactions in silico. In the present manuscript, we developed a mathematical model of material transfer between TGRL and HDL which simulated conditions of our in vitro experiments [25]. Consistent with our observations, mathematical modelling found an existence of a maximum at the dose-dependence of FC transfer to HDL, resulting from competitive fluxes of FC moving through the HDL pool.

## 2. Results

**Figure 1:**
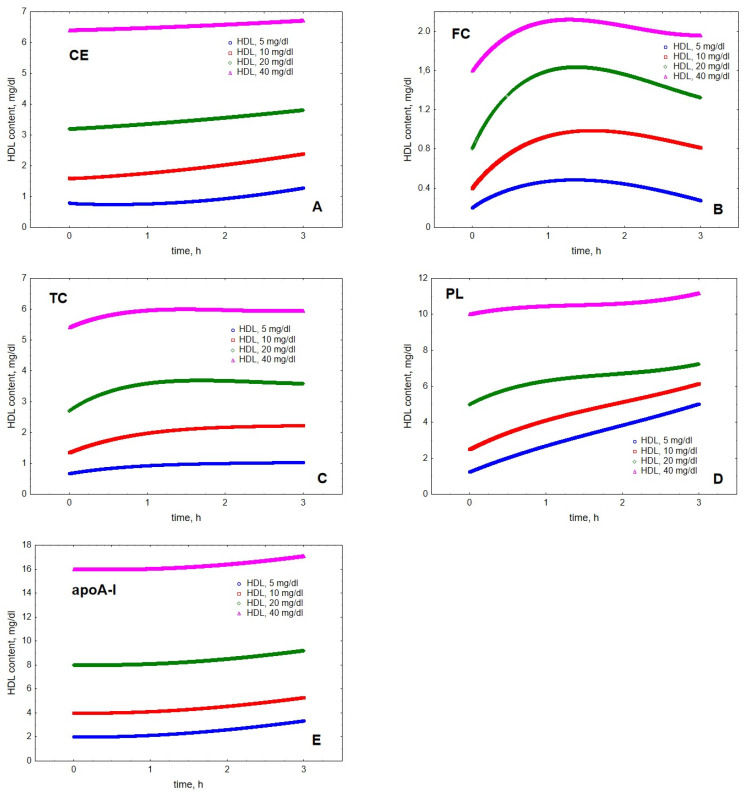
Kinetics of HDL content of CE (**A**), FC (**B**), TC (**C**), PL (**D**) and apoA-I (**E**) during VLDL lipolysis by LPL in the presence of HDL. Mathematical modelling was performed under conditions of in vitro TGRL lipolysis described elsewhere [25]. Initial concentrations: HDL, 5 (blue circles), 10 (red squares), 20 (green diamonds) and 40 (purple triangles) mg total mass/dL; VLDL-TG, 30 mg/dL; LPL, 190 U/mL.

**Figure 2:**
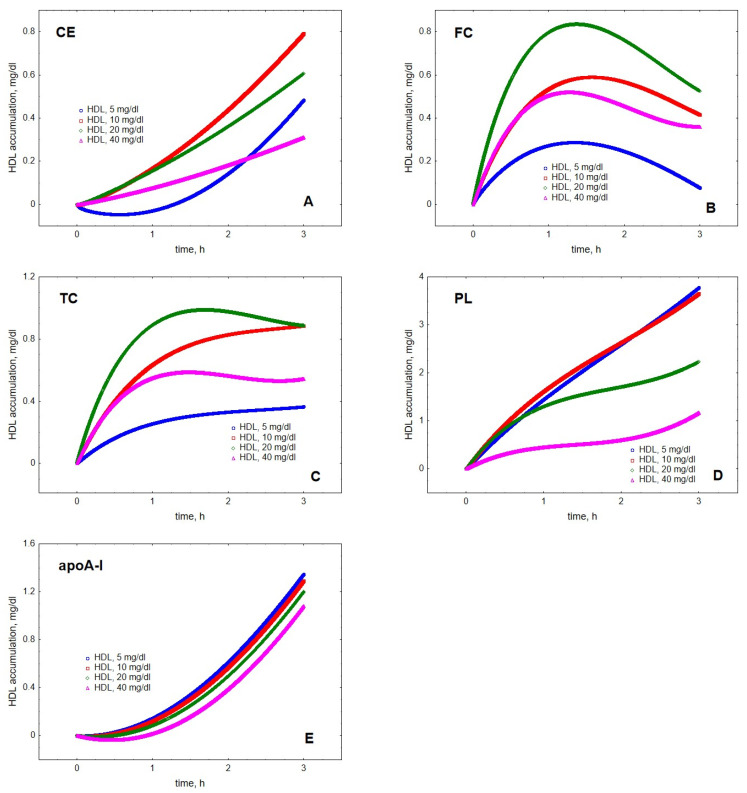
Kinetics of the accumulation in HDL of CE (**A**), FC (**B**), TC (**C**), PL (**D**) and apoA-I (**E**) during VLDL lipolysis by LPL in the presence of HDL. Mathematical modelling was performed under conditions of in vitro TGRL lipolysis described elsewhere [25]. In order to calculate accumulation in HDL of a given component, its content at baseline (t = 0) was subtracted from the results of the modelling. Initial concentrations: HDL, 5 (blue circles), 10 (red squares), 20 (green diamonds) and 40 (purple triangles) total mass/dL; VLDL-TG, 30 mg/dL; LPL, 190 U/mL.

Mathematical modelling of LPL-mediated TG lipolysis in VLDL in the presence of HDL revealed material transfer from VLDL to HDL. Indeed, cholesteryl ester (CE), FC, total cholesterol (TC), PL and apoA-I all accumulated in HDL over time (Figure 1). The transfer of all the components was time-dependent but the kinetics of material accumulation in HDL differed among individual lipoprotein components. Indeed, the kinetics of HDL content of FC was characterised by saturation over time and appearance of a maximum located between 1.2 and 1.6 h, which was observed at all HDL concentrations studied (Figure 1). In addition, HDL content of TC displayed an ambiguous maximum at HDL concentrations of 20 and 40 mg/dL, which was observed after 1.7 and 1.5 h, respectively. By contrast, HDL content of CE, PL and apoA-I steadily increased over the simulation time-frame of 3 h. The qualitatively distinct kinetics were even more obvious when HDL content of individual components was expressed as increments relative to baseline at the beginning of lipolysis (Figure 2). 

**Figure 3:**
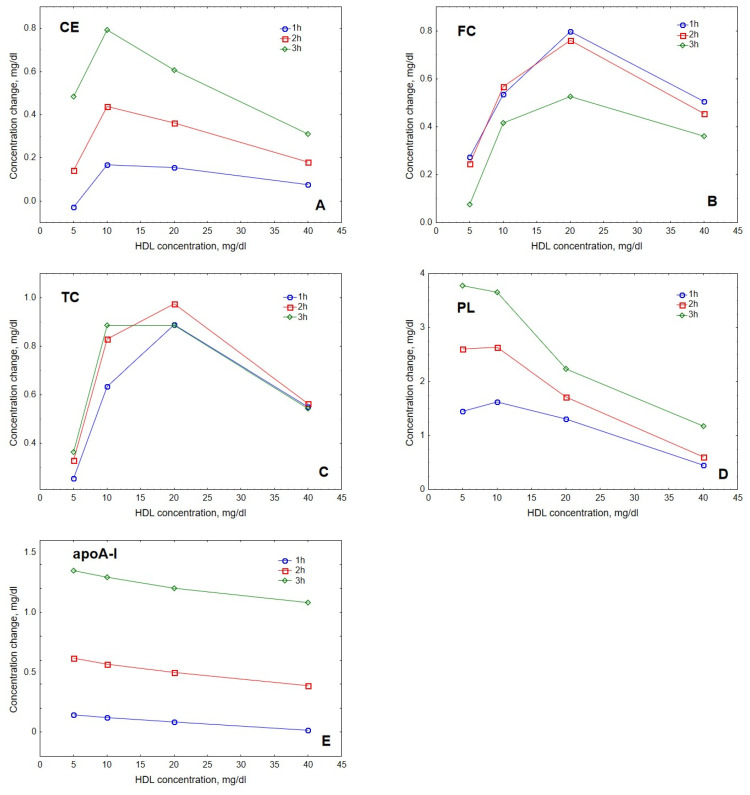
Dose-dependences of the accumulation in HDL of CE (**A**), FC (**B**), TC (**C**), PL (**D**) and apoA-I (**E**) during VLDL lipolysis by LPL in the presence of HDL. Mathematical modelling was performed under conditions of in vitro TGRL lipolysis described elsewhere [25] for the reaction time of 1 (blue circles), 2 (red squares) and 3 (green diamonds) h. In order to calculate accumulation in HDL of a given component, its content at baseline (t = 0) was subtracted from the results of the modelling. Initial concentrations: HDL, 5, 10, 20 and 40 mg total mass/dL; VLDL-TG, 30 mg/dL; LPL, 190 U/mL.

The modelling showed that the accumulation of lipids and protein in HDL depended on the concentration of acceptor HDL particles. Interestingly, HDL content of CE, FC and TC peaked at intermediate HDL concentrations of 10 to 20 mg/dL (Figure 3). Indeed, maximal CE accumulation in HDL of +0.8 mg/dL (+49%) relative to baseline was found at the intermediate HDL concentration of 10 mg/dL, whereas maximal FC and TC accumulation of +0.8 and +1.0 mg/dL (+104 and +37%, respectively) relative to baseline was observed at the HDL concentration of 20 mg/dL. By contrast, the dose-dependence of PL accumulation in HDL showed a weak maximum at 10 mg/dL, whereas the accumulation of apoA-I decreased over the whole concentration range studied.

**Figure 4:**
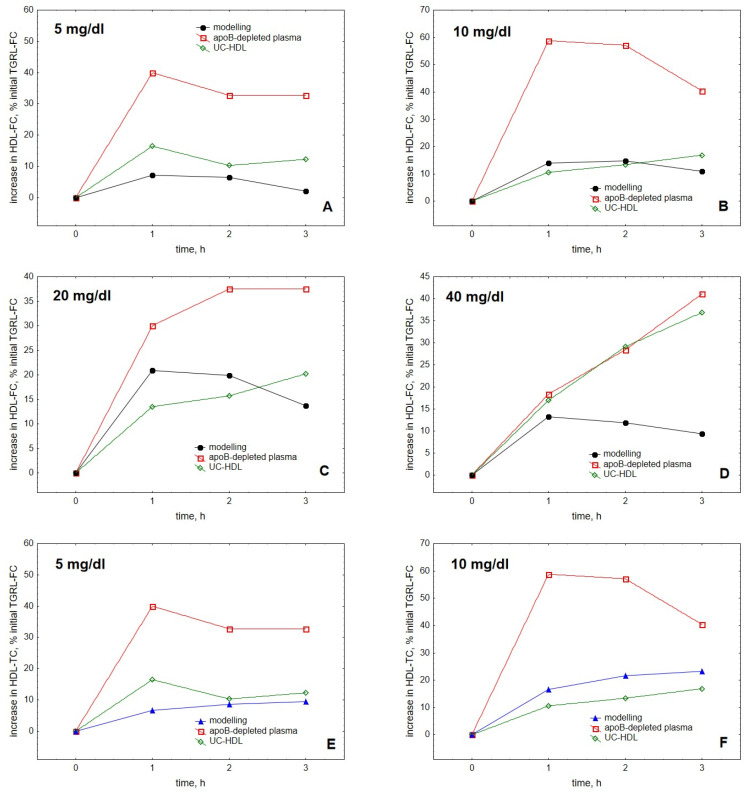

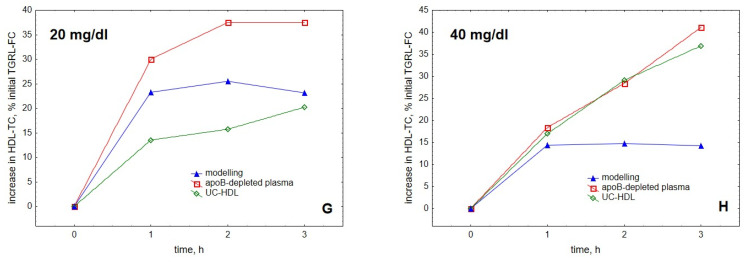
Comparison of the kinetics of accumulation in HDL of FC (**A**–**D**) and TC (**E**–**H**) with experimental data obtained for HDL isolated by ultracentrifugation or by apoB precipitation [25]. Experimental data for HDL isolated by ultracentrifugation and apoB precipitation are shown as green diamonds and red squares, respectively, whereas modelling results are presented as black circles for FC and blue triangles for TC. As the experimental data do not distinguish between TC and FC and are expressed relative to the initial content of FC in TGRL, all the data are expressed as a percentage of the initial TGRL-FC. Mathematical modelling was performed under conditions of in vitro TGRL lipolysis described elsewhere [25]. In order to calculate accumulation in HDL of FC or TC, its content at baseline (t = 0) was subtracted from the results of the modelling and expressed as a percentage of the initial VLDL content of FC, according to [25]. Initial concentrations: HDL, 5 (**A,E**), 10 (**B**,**F**), 20 (**C**,**G**) and 40 (**D**,**H**) mg total mass/dL (shown in the upper left corner of each panel); VLDL-TG, 30 mg/dL; LPL, 190 U/mL.

Comparison of the modelling results with experimentally obtained data showed that our model qualitatively well described cholesterol accumulation in HDL over time upon TGRL lipolysis by LPL observed in vitro [25] (Figure 4). Quantitative differences between the experimental and theoretical data may reflect both large inter-individual differences in the FC transfer and absence of knowledge of the distribution of the fluorescent label between free and esterified cholesterol as reported by us earlier [25].

The model was largely in agreement with the data reported for HDL obtained by both ultracentrifugation and apoB precipitation, better simulating the kinetics observed for ultracentrifugally isolated particles. Furthermore, the model was generally able to reproduce both the saturation of the curve of cholesterol accumulation in HDL over time (Figure 4) and the appearance of the maximum at this curve as a function of HDL concentration (Figure 3) [25].

**Figure 5:**
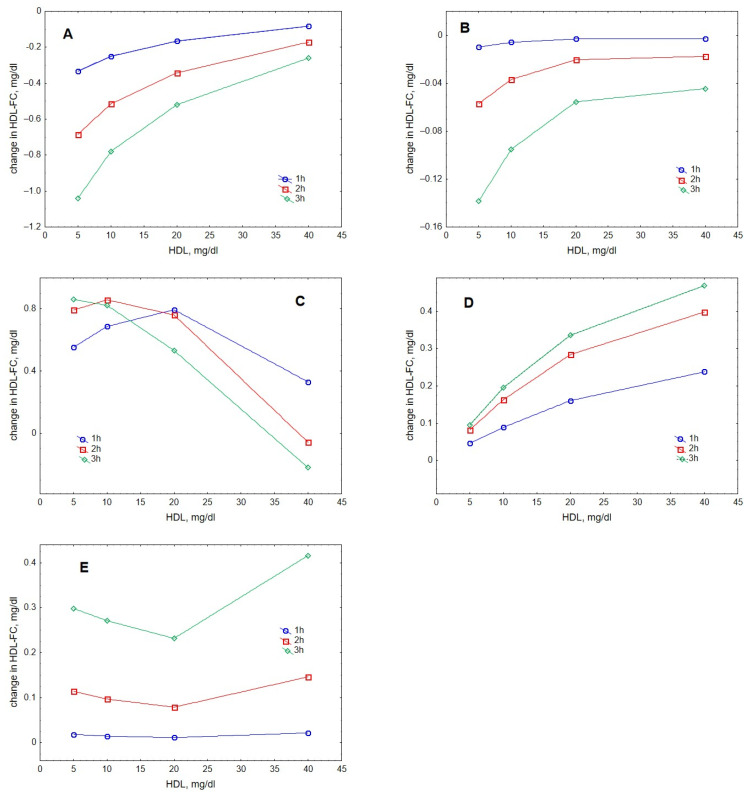
**Dose-dependences of contributions to HDL-FC provided by individual processes**, including LCAT-mediated formation of CE from FC in large, lipid-rich, spherical HDL (**A**), LCAT-mediated formation of CE from FC in small, lipid-poor, discoidal HDL (**B**), FC transfer from VLDL to HDL under the action of FC concentration gradient (**C**), FC transfer to HDL upon collisions between VLDL and HDL particles (**D**) and FC transfer to HDL upon fusion of lipid-poor apoA-I with lipid-rich HDL (**E**). Mathematical modelling was performed under conditions of in vitro TGRL lipolysis described elsewhere [25] for the reaction time of 1 (blue circles), 2 (red squares) and 3 (green diamonds) h. In order to calculate accumulation of FC in HDL, its content at baseline (t = 0) was subtracted from the results of the modelling. Initial concentrations: VLDL-TG, 30 mg/dL; LPL, 190 U/mL.

Analysis of individual processes regulating HDL content of FC suggested that lecithin-cholesterol acyltransferase (LCAT)-mediated esterification and gradient-driven transfer markedly influenced this metric, whereas contributions of collision- and fusion-mediated pathways were minor (Figure 5). Contributions of the reaction of esterification catalysed by LCAT increased with increasing HDL concentration when FC originated from either lipid-rich, spherical (Figure 5A) or lipid-poor, discoidal (Figure 5B) HDL. The input provided by lipid-rich particles was expectedly superior to that from lipid-poor HDL. By contrast, contributions of gradient-driven FC transfer from VLDL to HDL predominantly decreased with increasing HDL concentration, reflecting reduced FC concentration gradient between the lipoproteins (Figure 5C). Contributions from FC transfer upon particle collision tended to raise with increasing HDL concentrations (Figure 5D), whereas those from HDL particle fusion did not reveal a systematic dose-dependence (Figure 5E). Whatever the relationship, both processes contributed less to HDL-FC as compared to the gradient-driven transfer (except at the highest HDL concentration studied of 40 mg/dL when the contribution of the gradient-driven transfer was low).

**Figure 6:**
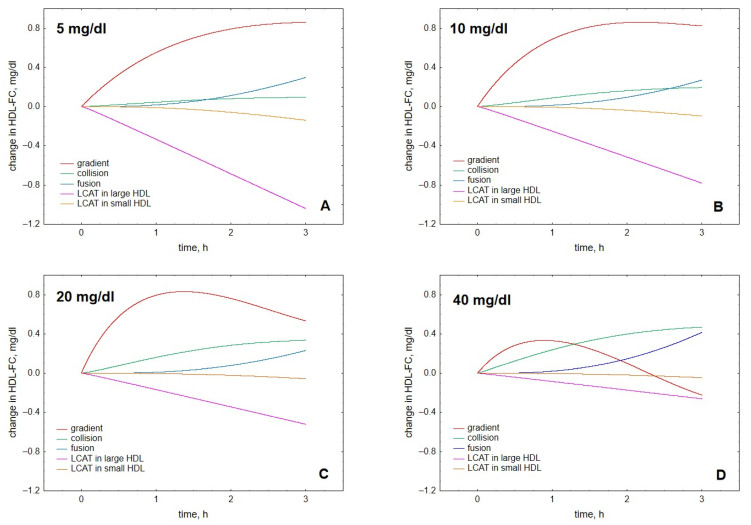
**Kinetics of contributions to HDL-FC provided by individual processes**, including LCAT-mediated formation of CE from FC in large, lipid-rich, spherical HDL (purple), LCAT-mediated formation of CE from FC in small, lipid-poor, discoidal HDL (brown), FC transfer from VLDL to HDL under the action of FC concentration gradient (red), FC transfer to HDL upon collisions between VLDL and HDL particles (green) and FC transfer to HDL upon fusion of lipid-poor and lipid-rich HDL (blue). Results are separately shown for HDL concentrations of 5 (**A**), 10 (**B**), 20 (**C**) and 40 (**D**) mg total mass/dL (indicated in the upper left corner of each panel). Mathematical modelling was performed under conditions of in vitro TGRL lipolysis described elsewhere [25]. In order to calculate accumulation of FC in HDL, its content at baseline (t = 0) was subtracted from the results of the modelling. Initial concentrations: VLDL-TG, 30 mg/dL; LPL, 190 U/mL.

**Figure 7:**
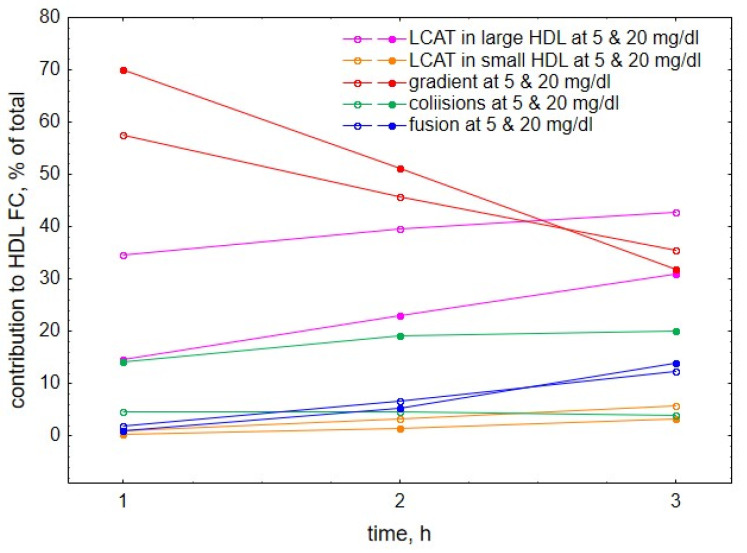
**Relative contributions to HDL-FC provided by individual processes**, including LCAT-mediated formation of CE from FC in large, lipid-rich, spherical HDL (purple), LCAT-mediated formation of CE from FC in small, lipid-poor, discoidal HDL (brown), FC transfer from VLDL to HDL under the action of FC concentration gradient (red), FC transfer to HDL upon collisions between VLDL and HDL particles (green) and FC transfer to HDL upon fusion of lipid-poor apoA-I with lipid-rich HDL (blue). All negative contributions were converted into positive and relative contributions of all processes were calculated as percentages of their sum. For the sake of simplicity, data are only shown for two HDL concentrations of 5 and 20 mg/dL. Mathematical modelling was performed under conditions of in vitro TGRL lipolysis described elsewhere [25]. In order to calculate accumulation of FC in HDL, its content at baseline (t = 0) was subtracted from the results of the modelling. Initial concentrations: VLDL-TG, 30 mg/dL; LPL, 190 U/mL.

Further comparison of the roles of the individual processes at different HDL concentrations showed that both at early reaction times (Figure 6A–D) and low HDL concentrations (Figure 6A,B), gradient-driven transfer from VLDL to HDL constituted the major source of HDL-FC, which was primarily counterbalanced by FC consumption during LCAT-mediated esterification. Indeed, we calculated that gradient-driven transfer and LCAT-mediated esterification contributed (with different signs) up to 70% and 43% of total cholesterol flux through HDL, respectively, whereas contributions from other processes were minor (Figure 7). The contribution of the gradient-driven transfer weakened with prolonging reaction time and increasing HDL concentration, whereas the contributions of collision- and fusion-mediated transfer raised, albeit less markedly (Figure 6). In parallel, HDL content of FC initially increased, then reached maximum and subsequently declined slightly (Figure 1 and Figure 3). 

**Figure 8:**
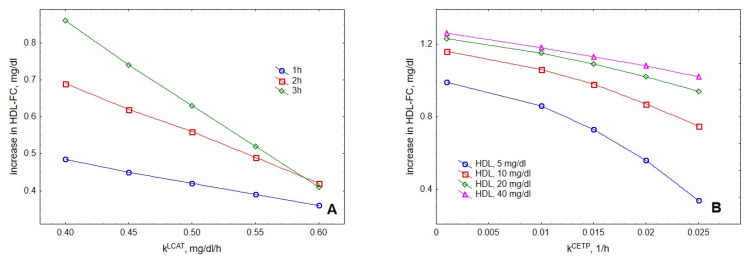
**Influence of LCAT (A) and CETP (B) activities on the accumulation of cholesterol in HDL.** Mathematical modelling was performed under conditions of in vitro TGRL lipolysis described elsewhere [25] for the reaction time of 1 (blue circles), 2 (red squares) and 3 (green diamonds) h (**A**). In order to calculate accumulation of cholesterol in HDL, its content at baseline (t = 0) was subtracted from the results of the modelling. Initial concentrations: VLDL-TG, 30 mg/dL; LPL, 190 U/mL. (**A**) HDL, 40 mg total mass/dL; (**B**) HDL, 5 (blue circles), 10 (red squares), 20 (green diamonds) and 40 (purple triangles) total mass/dL; reaction time, 2 h.

Evaluation of the roles of LCAT and CETP, two major proteins regulating HDL metabolism, showed that increasing their activities resulted in reduced cholesterol accumulation in HDL upon VLDL lipolysis (Figure 8). This result was consistent with experimental observations reported elsewhere [25].

## 3. Discussion

In the present manuscript, we developed an original mathematical model of material transfer to HDL upon VLDL lipolysis by LPL. The model was developed to simulate conditions of our in vitro experiments evaluating FC transfer from TGRL to HDL upon lipolysis [25] and included seven differential equations to describe kinetics of enzymatic reactions and mass transfer. Consistent with our observations, mathematical modelling revealed that cholesterol was accumulated in HDL upon lipolysis in parallel to the accumulation of other components of TGRL. Importantly, our simulation was able to reproduce major features of cholesterol accumulation in HDL observed experimentally, notably saturation of this process over time and appearance of a maximum as a function of HDL concentration [25].

Comparison of the modelling results with experimentally obtained data suggested that our model qualitatively well described cholesterol accumulation in HDL over time observed by us in vitro [25]. Indeed, calculated levels of FC and TC accumulation in HDL upon lipolysis were similar to those measured in HDL isolated by ultracentrifugation and slightly lower than those observed in HDL obtained by apoB precipitation. The latter approach isolates HDL together with plasma proteins which primarily include albumin [27]. As albumin can function as a low-affinity, high-capacity cholesterol transporter that enhances FC transfer to HDL during TGRL lipolysis [28,29], the presence of albumin in apoB-depleted samples may account for the elevated cholesterol accumulation as compared both to HDL isolated by ultracentrifugation [25] and to our mathematical modelling. It merits to be mentioned that in our experiments, we traced accumulation in HDL of fluorescent TopFluor^®^ cholesterol (also known as BODIPY cholesterol) which is efficiently esterified by LCAT and whose ester can be transferred by CETP [30,31]. In the absence of data on the distribution of the fluorescent label between free and esterified cholesterol [25], our experimentally observed kinetics should therefore be compared with the calculated kinetics of both FC and TC.

Our modelling further showed that although the accumulation in HDL of all studied lipoprotein components (CE, FC, TC, PL and apoA-I) was time-dependent, the kinetics of this process differed among them. Indeed, although the accumulation of FC and TC was characterised by saturation over time and presence of maximum, that of PL, CE and apoA-I steadily increased over time. Analysis of individual processes demonstrated that gradient-driven transfer and LCAT-mediated esterification provided key contributions to HDL content of FC, whereas the role of collision- and fusion-mediated pathways was minor. Our analysis suggested that the appearance of the maximum at the kinetics of FC accumulation in HDL resulted from the existence of competitive fluxes of FC moving through the HDL pool. More precisely, gradient-driven FC transfer from TGRL to HDL provided a major contribution to HDL-FC at early reaction times when it was only partially counterbalanced by FC consumption during LCAT-mediated esterification. At this stage, the role of other kinetic processes was minor. At later reaction times, FC accumulation in HDL led to a reduced concentration gradient between TGRL and HDL, resulting in the saturation of the gradient-driven FC transfer and appearance of the maximum secondary to enhanced LCAT-mediated esterification; the latter equally reflected FC accumulation in HDL. As LCAT-mediated esterification was coupled to CE removal from HDL by CETP, it was not saturated over time. In the absence of a similar mechanism for the removal from HDL of PL and apoA-I, no maximum was observed for the kinetics of HDL content of these components. In turn, the accumulation of CE in HDL was governed by the ratio of the rates of CE formation by LCAT and CE removal by CETP and did not produce kinetic maximum, further emphasising the distinct nature of the regulation of FC metabolism. As a corollary, the kinetics of TC accumulation in HDL followed those of FC.

Our modelling further showed that the accumulation of lipids and protein in HDL was dose-dependent. Indeed, HDL content of cholesterol initially increased with increasing HDL concentrations and subsequently fell at high HDL concentrations. Similar to the considerations above, a competition between influx of FC into and efflux of FC from HDL could account for this observation. Indeed, influx of FC provided by the gradient-driven transfer from TGRL was reduced at high HDL concentrations, whereas efflux of FC via collision-mediated transfer was predominantly raised, albeit less markedly. Combined with the consumption of FC by LCAT-mediated esterification, these pathways resulted in the appearance of the maximum at the dose-dependent curve of HDL-FC. By contrast, the more straightforward nature of the transfer of PL and apoA-I was associated with predominantly linear relationships between their accumulation in HDL and HDL concentrations in the reaction mixture, primarily reflecting reduced gradient-driven transfer from TGRL to HDL at high HDL concentrations. 

Consistent with the mechanisms outlined above, evaluation of the roles of LCAT and CETP demonstrated that cholesterol accumulation in HDL upon TGRL lipolysis was decreased when activities of LCAT and CETP were elevated. This result is in agreement with enhanced accumulation of cholesterol in HDL upon TGRL lipolysis observed in the presence of inhibitors for LCAT or CETP [25] and can be explained by a multi-step mechanism of FC movement between HDL and TGRL upon LPL-induced lipolysis, which involves acquirement of FC by HDL with its subsequent esterification by LCAT followed by CETP-mediated exchange of the generated CE for a TG present in TGRL. Importantly, such decreased cholesterol accumulation in HDL observed when LCAT and CETP activities are elevated should not necessarily be interpreted as delayed cholesterol removal from circulation in vivo as cholesterol transferred through this pathway to apoB-containing lipoproteins typically is efficiently cleared via LDL-receptors, representing quantitatively major pathway of cholesterol clearance from plasma in humans [32].

Together with our experimental observations [25], results of our mathematical modelling further support the RRT hypothesis recently developed by us [26,29]. The RRT hypothesis was advanced in order to provide explanation for the U-shaped relationship between HDL-C and cardiovascular disease [10,11,12]. Impaired transfer to HDL of FC released during TGRL lipolysis in subjects with both low and extremely high HDL-C levels was hypothesised to account for the U-shaped epidemiology. Both experimental measurements and mathematical modelling report that accumulation of FC in HDL upon TGRL lipolysis by LPL is reduced when HDL concentrations are either low or extremely high, peaking at intermediate concentrations which are optimal for this process. Our present study sheds more light on the mechanisms potentially underlying this seemingly paradoxical [12] phenomenon. We found that the existence of the maximum at the dose-dependence of FC accumulation in HDL results from the competitive fluxes of FC through the HDL pool, including those driven by FC concentration gradient between TGRL and HDL on the one hand and those mediated by LCAT and CETP on the other hand. More specifically, FC accumulation in HDL decreases at high HDL concentrations as a result of a reduced concentration gradient between TGRL and HDL, with extremely high HDL concentrations impairing further FC acquirement by HDL. 

Our findings provide novel opportunities to revisit our view of HDL in the framework of the RRT hypothesis. If acquirement of surface TGRL remnants with subsequent transport of their FC to the liver does constitutes the major biological activity of HDL [26,29,33], new therapies can then be developed in order to normalise this process in subjects with both low and extremely high HDL-C levels. In low HDL-C subjects, such approaches may primarily involve activation of TGRL lipolysis by LPL coupled to moderate HDL-C-raising; the latter can be achieved, e.g., via accelerated hepatic secretion of apoA-I and/or enhanced lipidation of apoA-I upon interaction with ABC transporters. When HDL-C is extremely high, accelerated removal of HDL-derived cholesterol from circulation may prove useful (e.g., via enhanced function of scavenger receptor BI). As a result, efficient cholesterol transport through the HDL pool to the liver with excretion into the bile may reduce influx in the arterial wall of atherogenic FC derived from TGRL, normalising HDL-C levels and delaying atherogenesis.

## 4. Methods

### 4.1. Model Structure

We aimed at developing a mathematical model of lipid and protein transfer between TGRL and HDL upon TGRL lipolysis by LPL in vitro. We attempted to properly describe key metabolic processes while keeping the model as simple as possible. Our purpose was to compare the kinetics of HDL alterations observed in vitro [25] with those calculated in silico using the model. 

#### 4.1.1. Lipoprotein Representation

TGRLs typically include chylomicrons, VLDL and intermediate-density lipoproteins (IDL); as chylomicrons are rapidly catabolised and IDL are typically present in plasma at low concentrations, we only included VLDL in the model as a representative TGRL particle. As HDL particles are heterogeneous in physicochemical, compositional and biological properties [34], the behaviour of two major HDL subclasses was modelled in the present study, which included large, lipid-rich, spherical alpha-HDL and small, lipid-poor, discoidal pre-beta apoA-I (Alp) particles. Lipoproteins were assumed to be composed of CE, TG, FC, PL and protein. TC was calculated as the sum of FC and cholesterol moiety of CE according to the relationship $TC_{\alpha }=CE_{\alpha }\cdot 0.595+FC_{\alpha }$ [35,36]. Lipoproteins and their components included in the model are shown in Table 1.

#### 4.1.2. Metabolic Processes

During LPL-mediated lipolysis, TGRL content of TG is decreased. As TGRL core is predominantly composed of TGs, its size is reduced concomitant with the decrease in TG. When a TGRL particle shrinks, and its surface components (PL, FC and surface proteins) become excessive, they cannot be retained by the smaller core and are detached from the particle. As a result, surface lipids and proteins derived from TGRL upon lipolysis form surface TGRL remnants which subsequently fuse with large HDL, or form small, lipid-poor HDL, contributing to the HDL pool [16,17,18].

The model developed to describe these processes therefore included VLDL, large, lipid-rich HDL and small, lipid-poor HDL as three lipoprotein classes; the latter was assumed to only contain apoA-I, PL and FC and was therefore termed lipid-poor apoA-I (Alp). In addition, the model took into account other processes of HDL remodelling upon VLDL lipolysis as follows: (i) heteroexchange of TG and CE between VLDL and HDL mediated by CETP; (ii) exchange of CE between VLDL and HDL mediated by phospholipid transfer protein (PLTP); (iii) conversion of FC to CE in HDL under the action of LCAT; (iv) exchange of FC between VLDL and HDL as a result of FC concentration gradient; (v) exchange of FC between VLDL and HDL upon collision of lipoproteins during their diffusion; (vi) exchange of PL across VLDL and HDL as a result of PL concentration gradient; (vii) exchange of PL between VLDL and HDL upon collision of lipoproteins during their diffusion; (viii) exchange of PL across VLDL and HDL under the action of PLTP; (ix) fusion of lipid-poor apoA-I with HDL; and (x) release of lipid-poor apoA-I from HDL via dissociation.

**Figure 9:**
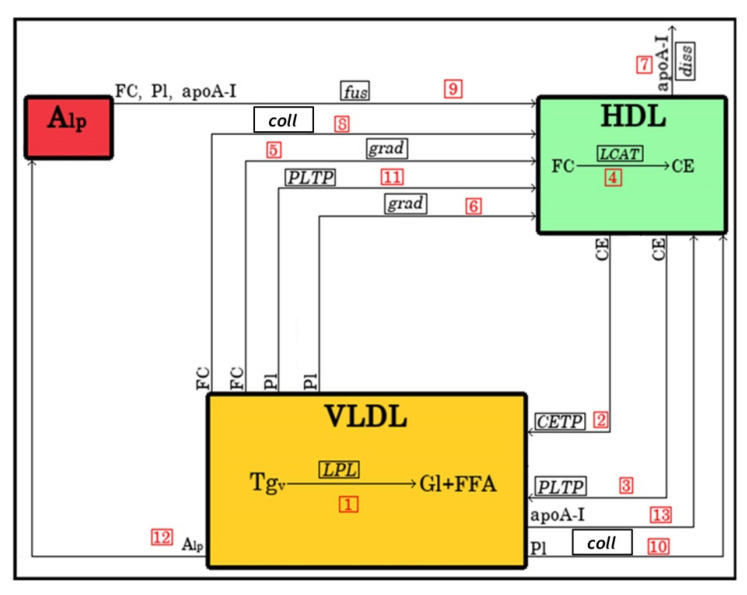
**Schematic representation ofthe model oflipoprotein remodellingupon VLDL lipolysis by LPL in the presence of HDL**. The arrows shown represent processes described in the text which are equally abbreviated in italics. The arrows are numbered according to the reaction numbers shown in Table 2. Gl, glycerol; FFA, free fatty acids; grad, gradient; diss, dissociation; coll, collision.

The initiating process included in the model (Figure 9) involved decomposition of TG in VLDL under the action of LPL to glycerol and free fatty acids (FFA; arrow 1) with ensuing transfer of FC, PL and surface proteins (represented by apoA-I alone for the sake of simplicity) from VLDL to HDL. Accumulation of CE in HDL was modelled to result from the transfer of CE from HDL to VLDL by CETP and PLTP (arrows 2 and 3, respectively) as well as from LCAT-mediated conversion to CE of FC both present in HDL (arrow 4) and derived from lipid-poor apoA-I upon its fusion with HDL (arrow 9). Transfer to HDL of PL was ensured by PLTP (arrow 11). The model also took into account exchange of FC and PL under the action of their concentration gradients between VLDL and HDL (arrows 5 and 6, respectively) as well as dissociation of apoA-I from HDL (arrow 7). Furthermore, the exchange of FC and PL resulting from collisions between VLDL and HDL was included in the model (arrows 8 and 10, respectively). Finally, fusion with HDL of VLDL-derived lipid-poor apoA-I resulted in the transfer to HDL of FC, PL and apoA-I (arrow 12); in addition, lipid-free apoA-I was transferred to HDL in a separate pathway (arrow 13). 

In order to precisely model these processes, detailed knowledge of their main characteristics, including rate constants, diffusion coefficients, viscosity, thermal conductivity and others, is required; these data are however largely absent from the literature. For this reason, we modelled the exchange and transfer processes as first-order concentration-dependent kinetics, consistent with established first-order kinetics of FC and PL transfer across lipoproteins [37,38,39]. When rate constants were unknown, they were modelled as time- or concentration-dependent parameters as long as such dependences were established elsewhere. A complete list of chemical reactions and transfer processes included in the model is shown in Table 2, together with expressions of their reaction rates as detailed below.

### 4.2. Mathematical Description of the Model

VLDL lipolysis under the action of LPL occurs via breakdown of TG molecules present in the VLDL core with a consecutive formation of diglycerides, monoglycerides, FFA and glycerol. Moate et al. [40] described the lipolysis process by the following set and auxiliary system of differential equations:

$$\frac{dTg}{dt}=-\frac{k_{1}\cdot Tg\left(t\right)}{k_{2}+Tg\left(t\right)}$$

$$\frac{dDg}{dt}=\frac{2\cdot k_{1}\cdot Tg\left(t\right)}{3\cdot \left(k_{2}+Tg\left(t\right)\right)}$$

$$\frac{dMg}{dt}=\frac{k_{3}\cdot Dg\left(t\right)}{2}-k_{3}\cdot Mg\left(t\right)$$

(1) $$\frac{d\left(FFA+Gl\right)}{dt}=\frac{k_{1}\cdot Tg\left(t\right)}{3\cdot \left(k_{2}+Tg\left(t\right)\right)}+\frac{k_{3}\cdot Dg\left(t\right)}{2}+k_{3}\cdot Mg\left(t\right)$$

where *Tg*(*t*), *Dg*(*t*), *Mg*(*t*), *FFA*(*t*) and *Gl*(*t*) are the concentrations of TG, diglycerides, monoglycerides, *FFA* and glycerol, respectively. The constant *k*_1_ [(mg/dL)/h] is the maximum reaction rate, *k*_2_ is the Michaelis constant equal to the concentration of TG [mg/dL] at which the reaction rate is half of its maximum value, and *k*_3_ [1/h] is a first-order rate constant describing lipolysis of mono- and diglycerides.

Consistent with this approach, we assumed that the decomposition of VLDL-TG by LPL during lipolysis can be described by the following equation:

(2) $$\frac{dTg_{V}\left(t\right)}{dt}=\frac{V_{Tg}^{max}\cdot Tg_{V}\left(t\right)}{k_{Tg}^{m}+Tg_{V}\left(t\right)}$$

where *Tg_V_*(*t*) is the concentrations of TG in VLDL, *V^max^_Tg_* is the maximum reaction rate and *k^m^_Tg_* is the Michaelis constant.

In addition to TG, the core of VLDL equally contains CE which is accumulated by VLDL over time as a result of CETP activity according to the equation:

(3) $$\frac{dCE_{V}\left(t\right)}{dt}=k^{CETP}\left(t\right)\cdot CE_{\alpha }\left(t\right)$$

where *k^CETP^* is the rate constant of CETP-mediated heteroexchange of CE and TG between VLDL and HDL, and *CE_V_* and *CE_α_* are CE concentrations in VLDL and HDL, respectively. In this equation, *k^CETP^* does not remain constant over time, reflecting the observation that the rate of CETP-mediated heteroexchange of CE and TG depends on lipoprotein composition [41]. Indeed, relative depletion of CE occurring upon TG enrichment increases rates of fusion and dissociation of HDL during their remodelling, leading to enhancement of CETP activity [41]. Then the rate constant of CETP-mediated heteroexchange of CE and TG should also depend on the increase in TG concentration in the HDL core as follows:

(4) $$k^{CETP}\left(t\right)=k^{CETP}\cdot p_{1}(t)$$

where *p*_1_(*t*) is the relative increase of TG content in HDL defined as the sum of the initial HDL content of TG and the enrichment of HDL in TG as a result of CETP activity divided by the initial HDL content of TG. According to Cobbold et al. [42], the number of TG molecules transferred from VLDL to HDL is equal to the number of CE molecules transferred from HDL to VLDL during their heteroexchange. Therefore, the enrichment of large alpha-HDL in TG as a result of CETP activity (*Tg_α_*) can be defined as $\left[\frac{\%Tg_{\alpha }}{\%CE_{\alpha }}\cdot \frac{m_{Tg}}{m_{CE}}\cdot CE_{V}\left(t\right)\right]$, where %*Tg_α_* and %*CE_α_* are wt% of TG and CE in HDL, respectively, and *m_Tg_* and *m_CE_* are average molar masses of TG and CE molecules, respectively. Then, the relative increase of TG content in HDL can be expressed according to the following equation:

(5) $$p_{1}\left(t\right)=\frac{Tg_{\alpha_{0}}+\left[\frac{\%Tg_{\alpha }}{\%CE_{\alpha }}\cdot \frac{m_{Tg}}{m_{CE}}\cdot CE_{V}\left(t\right)\right]}{Tg_{\alpha_{0}}}$$

where *Tg_α_*_0_ is the initial TG content in HDL. 

Similar to VLDL, the core of HDL contains CE and TG molecules. CE content of HDL (*CE_α_*) is determined by CE removal under the action of CETP as well as by CE formation during esterification of FC in the LCAT reaction. CE removal from HDL by CETP is equal (with a negative sign) to CE enrichment of VLDL. FC used for the LCAT reaction comprises FC initially present in HDL and FC brought to HDL by the fusion with lipid-poor particles (Alp) generated upon VLDL lipolysis. These considerations resulted in the following equations for CE produced in alpha-HDL by the LCAT reaction according to Michaelis–Menten kinetics (*CE_α__MM_*):

$$\frac{dCE_{\alpha_{MM}}}{dt}=\frac{k^{LCAT}\cdot \left[Tg_{V_{0}}-Tg_{V}\left(t\right)\right]\cdot \frac{\%FC_{V}}{\%Tg_{V}}\cdot \frac{S_{V}}{S_{k_{V}}}\cdot \frac{m_{FC}}{m_{Tg}}}{k_{FC}^{m}+\left[Tg_{V_{0}}-Tg_{V}\left(t\right)\right]\cdot \frac{\%FC_{V}}{\%Tg_{V}}\cdot \frac{S_{V}}{S_{k_{V}}}\cdot \frac{m_{FC}}{m_{Tg}}};$$

(6) $$\frac{dCE_{\alpha_{fus}}}{dt}=\frac{k^{LCAT}\cdot \frac{k^{fus}}{\left(A_{\alpha }\left(t\right)/A_{\alpha_{0}}\right)}\cdot A_{lp}\left(t\right)\cdot \frac{5\cdot m_{FC}}{m_{A_{lp}}}}{k_{FC}^{m}+\frac{k^{fus}}{\left(A_{\alpha }\left(t\right)/A_{\alpha_{0}}\right)}\cdot A_{lp}\left(t\right)\cdot \frac{5\cdot m_{FC}}{m_{A_{lp}}}}.$$

We further denoted $\left[Tg_{V_{0}}-Tg_{V}\left(t\right)\right]\cdot \frac{\%FC_{V}}{\%Tg_{V}}\cdot \frac{S_{V}}{S_{c_{V}}}\cdot \frac{m_{FC}}{m_{Tg}}=FC_{V}^{rem}\left(t\right)$ as the removal of FC from VLDL to HDL upon lipolysis, where *Tg_V_*_0_ and *Tg_V_*(*t*) are TG concentrations in VLDL at the beginning of the reaction and at a given time-point t, respectively, %*FC_V_* and %*Tg_V_* are wt% of FC and TG in VLDL, respectively, *S_V_* and *S_cV_* are surface area and core surface area of VLDL, respectively and *m_FC_* and *m_Tg_* are average molar masses of FC and TG, respectively. In addition, we denoted $\frac{k^{fus}}{\left(A_{\alpha }\left(t\right)/A_{\alpha_{0}}\right)}\cdot A_{lp}\left(t\right)=k^{fus}\left(t\right)\cdot A_{lp}\left(t\right)$ as the fusion of lipid-poor HDL particles with mature alpha-HDL followed by the transfer of FC, apoA-I and PL to alpha-HDL.

Next, we assumed that the lipid-poor HDL particles contained 5 FC molecules, 50 PL molecules and 1 apoA-I molecule [43]. Based on this, we obtained the following expressions:

$\frac{k^{fus}}{\left(A_{\alpha }\left(t\right)/A_{\alpha_{0}}\right)}\cdot \frac{5\cdot m_{FC}}{m_{A_{lp}}}\cdot A_{lp}\left(t\right)=FC_{Alp}^{fus}\left(t\right)$—concentration of FC transferred from Alp to HDL as a result of their fusion;

$\frac{k^{fus}}{\left(A_{\alpha }\left(t\right)/A_{\alpha_{0}}\right)}\cdot \frac{50\cdot m_{Pl}}{m_{A_{lp}}}\cdot A_{lp}\left(t\right)=Pl_{Alp}^{fus}\left(t\right)$—concentration of PL transferred from Alp to HDL as a result of their fusion;

$\frac{k^{fus}}{\left(A_{\alpha }\left(t\right)/A_{\alpha_{0}}\right)}\cdot \frac{m_{apoA-I}}{m_{A_{lp}}}\cdot A_{lp}\left(t\right)=apoA-I_{Alp}^{fus}\left(t\right)$—concentration of apoA-I transferred from Alp to HDL as a result of their fusion. 

Whereas PLTP has no intrinsic CETP activity, it enhances the transfer of CE from HDL to VLDL [44]. This effect was added to the model at a rate expressed as $k_{CE}^{PLTP}\cdot CE_{\alpha }\left(t\right)$. In the absence of precise kinetic data, we assumed the rate constant of PLTP-enhanced CE transfer *k^PLTP^_CE_* to be an order of magnitude lower than the rate constant of CETP-mediated CE transfer *k^CETP^_CE_* at low HDL concentrations and two orders of magnitude lower at high HDL concentrations. 

Putting together all the terms which contribute to HDL CE (*CE_α_*), we obtained the following equation:

(7) $$\frac{dCE_{\alpha }\left(t\right)}{dt}=-k^{CETP}\left(t\right)\cdot CE_{\alpha }\left(t\right)+\frac{k^{LCAT}\cdot FC_{V}^{rem}\left(t\right)}{k_{FC}^{m}+FC_{V}^{rem}\left(t\right)}+\frac{k^{LCAT}\cdot FC_{Alp}^{fus}\left(t\right)}{k_{FC}^{m}+FC_{Alp}^{fus}\left(t\right)}-k_{CE}^{PLTP}\cdot CE_{\alpha }\left(t\right).$$

To calculate HDL content of FC, FC conversion to CE under the action of LCAT should primarily be taken into account, which provides negative contribution to HDL FC and depends on the HDL FC/CE ratio. Furthermore, FC is transferred across lipoproteins upon their collision as well as under the influence of FC concentration gradient between VLDL and HDL, which is governed by the concentrations of FC in these particles (*FC_α_* and *FC_V_*). These processes were represented in the form of first-order kinetics whose rate constants depended on the magnitude and sign of the concentration gradient as follows:

(8) $$\left[\frac{FC_{V}\left(t\right)-FC_{\alpha }\left(t\right)}{FC_{V}\left(t\right)+FC_{\alpha }\left(t\right)}\right]=grad_{FC}^{rel};\;k_{FC}^{grad}\left(t\right)=k_{FC}^{grad}\cdot grad_{FC}^{rel}.$$

As a corollary, the contribution to HDL FC of the gradient transfer of FC between HDL and VLDL was expressed as $k_{FC}^{grad}\left(t\right)\cdot FC_{V}\left(t\right)$.

In addition, contributions to lipid transfer of collisions between VLDL and HDL were taken into account. These processes were represented as second-order kinetics in the form of $k_{FC}^{coll}\cdot FC_{\alpha }\left(t\right)\cdot FC_{V}\left(t\right)$. The transfer of FC from lipid-poor apoA-I to HDL was described in the same way as the transfer of CE described above. 

As a result, the equation for the time-dependence of FC content of HDL was as follows:

$$\frac{dFC_{\alpha }\left(t\right)}{dt}=k_{FC}^{grad}\left(t\right)\cdot FC_{V}\left(t\right)+k_{FC}^{coll}\cdot FC_{\alpha }\left(t\right)\cdot FC_{V}\left(t\right)+FC_{Alp}^{fus}\left(t\right)-$$

(9) $$-\left[\frac{k^{LCAT}\cdot FC_{V}^{rem}\left(t\right)}{k_{FC}^{m}+FC_{V}^{rem}\left(t\right)}\right]\cdot \frac{m_{FC}}{m_{CE}}-\left[\frac{k^{LCAT}\cdot FC_{Alp}^{fus}\left(t\right)}{k_{FC}^{m}+FC_{Alp}^{fus}\left(t\right)}\right]\cdot \frac{m_{FC}}{m_{CE}}$$

Next, concentrations of apoA-I in both lipid-poor HDL particles (Alp) and mature alpha-HDL were calculated. Production of lipid-poor apoA-I from VLDL upon lipolysis was represented as:

(10) $$A_{lp}^{rem}\left(t\right)=\left(Tg_{V_{0}}-Tg_{V}\left(t\right)\right)\cdot \frac{\%A_{V}}{\%Tg_{V}}\cdot \frac{S_{V}}{S_{c_{V}}}\cdot \frac{m_{A_{lp}}}{m_{apoA-I}}\cdot \frac{m_{apoA-I}}{m_{Tg}}.$$

Taking into account that lipid-poor apoA-I was consumed upon its fusion with HDL, we obtained:

(11) $$\frac{dA_{lp}\left(t\right)}{dt}=A_{lp}^{rem}\left(t\right)-k^{fus}\left(t\right)\cdot A_{lp}\left(t\right)$$

In a similar fashion, concentration of apoA-I in alpha-HDL was calculated as follows:

(12) $$\frac{dA_{\alpha }\left(t\right)}{dt}=apoA-I_{Alp}^{fus}\left(t\right)-k^{diss}\left(t\right)\cdot A_{\alpha }\left(t\right)+A_{V}^{rem}\left(t\right),$$

where the term $k^{diss}\left(t\right)=k^{diss}\cdot \frac{Pl_{\alpha }\left(t\right)}{Pl_{\alpha_{0}}}$ represented dissociation of apoA-I from HDL and the term $A_{V}^{rem}\left(t\right)=\left(Tg_{V_{0}}-Tg_{V}\left(t\right)\right)\cdot \frac{\%A_{V}}{\%Tg_{V}}\cdot \frac{S_{V}}{S_{c_{V}}}\cdot \frac{m_{apoA-I}}{m_{Tg}}$ represented transfer of apoA-I from VLDL to HDL. The rate of dissociation of apoA-I was assumed to be determined by alterations of the concentration of PL in alpha-HDL, whereas the rate of fusion was determined by alterations in the concentration of apoA-I.

Finally, differential equation for the time-dependence of PL concentration in alpha-HDL was written as:

(13) $$\frac{dPl_{\alpha }\left(t\right)}{dt}=k_{Pl}^{grad}\left(t\right)\cdot Pl_{V}\left(t\right)+k_{Pl}^{coll}\cdot Pl_{\alpha }\left(t\right)\cdot Pl_{V}\left(t\right)+Pl_{Alp}^{fus}\left(t\right)+k_{Pl}^{PLTP}\cdot Pl_{V}^{rem}\left(t\right),$$

where $Pl_{V}^{rem}\left(t\right)=\left[Tg_{V_{0}}-Tg_{v}\left(t\right)\right]\cdot \frac{\%Pl_{V}}{\%Tg_{V}}\cdot \frac{S_{V}}{S_{c_{V}}}\cdot \frac{m_{Pl}}{m_{Tg}}$ stayed for the removal of PL from VLDL to HDL upon lipolysis.

As a result, the model which included the reactions shown in Table 2 was represented by a system of seven first-order differential equations as follows:

$$\frac{dTg_{V}\left(t\right)}{dt}=\frac{V_{Tg}^{max}\cdot Tg_{V}\left(t\right)}{k_{Tg}^{m}+Tg_{V}\left(t\right)};$$

$$\frac{dCE_{V}\left(t\right)}{dt}=k^{CETP}\left(t\right)\cdot CE_{\alpha }\left(t\right);$$

$$\frac{dCE_{\alpha }\left(t\right)}{dt}=-k^{CETP}\left(t\right)\cdot CE_{\alpha }\left(t\right)+\frac{k^{LCAT}\cdot FC_{V}^{rem}\left(t\right)}{k_{FC}^{m}+FC_{V}^{rem}\left(t\right)}+\frac{k^{LCAT}\cdot FC_{Alp}^{fus}\left(t\right)}{k_{FC}^{m}+FC_{Alp}^{fus}\left(t\right)}-k_{CE}^{PLTP}\cdot CE_{\alpha }\left(t\right);$$

$$\frac{dFC_{\alpha }\left(t\right)}{dt}=k_{FC}^{grad}\left(t\right)\cdot FC_{V}\left(t\right)+k_{FC}^{coll}\cdot FC_{\alpha }\left(t\right)\cdot FC_{V}\left(t\right)+FC_{Alp}^{fus}\left(t\right)-$$

$$-\left[\frac{k^{LCAT}\cdot FC_{V}^{rem}\left(t\right)}{k_{FC}^{m}+FC_{V}^{rem}\left(t\right)}\right]\cdot \frac{m_{FC}}{m_{CE}}-\left[\frac{k^{LCAT}\cdot FC_{Alp}^{fus}\left(t\right)}{k_{FC}^{m}+FC_{Alp}^{fus}\left(t\right)}\right]\cdot \frac{m_{FC}}{m_{CE}};$$

$$\frac{dA_{lp}\left(t\right)}{dt}=A_{lp}^{rem}\left(t\right)-k^{fus}\left(t\right)\cdot A_{lp}\left(t\right);$$

$$\frac{dA_{\alpha }\left(t\right)}{dt}=apoA-I_{Alp}^{fus}\left(t\right)-k^{diss}\left(t\right)\cdot A_{\alpha }\left(t\right)+A_{V}^{rem}\left(t\right);$$

$$\frac{dPl_{\alpha }\left(t\right)}{dt}=k_{Pl}^{grad}\left(t\right)\cdot Pl_{V}\left(t\right)+k_{Pl}^{coll}\cdot Pl_{\alpha }\left(t\right)\cdot Pl_{V}\left(t\right)+Pl_{Alp}^{fus}\left(t\right)+k_{Pl}^{PLTP}\cdot Pl_{V}^{rem}\left(t\right).$$

From a mathematical point of view, solution for this system requires knowledge of all the parameters, initial concentrations of all the components and kinetic parameters, which are given in Table 3, Table 4, Table 5 and Table 6 and calculated based on data from [1,25,45,46,47,48,49,50]. The system was solved using the MATLAB software package over reaction time of up to 3 h which was chosen to compare modelling results with experimentally obtained data [25].

Lipoprotein composition was from Shen et al. [45]. Total concentration of VLDL-TG was fixed at 30 mg/dL [25], corresponding to 2.76 mg FC/dL, 6.76 mg PL/dL, 5.51 mg CE/dL and 2.4 mg apoA-I/dL. Since lipid-poor apoA-I (Alp) is formed during lipolysis, its initial concentration is zero. 

The values of the rate constants were obtained from published data [1,25,45,46,47,48,49,50]. For some processes, corrections were made to take into account variations of process rates as a function of HDL concentration as follows: CETP-mediated transfer, 1.0, 0.8, 0.6 and 0.4; LCAT-mediated esterification, 1.0, 0.75, 0.50 and 0.25; HDL particle fusion, 1.0, 3.3, 10 and 33; gradient-driven FC transfer, 1.0, 1.7, 2.3 and 3.3; gradient-driven PL transfer, 1.0, 1.3, 2.0 and 3.3, and apoA-I dissociation from HDL, 1.0, 1.0, 2.0 and 2.0 for HDL concentrations of 5, 10, 20 and 40 mg/dL, respectively.

### 4.3. Variational Analysis

As existing knowledge of the rate constants employed in the model is limited, we evaluated their influence on the kinetics of lipid accumulation in HDL upon VLDL lipolysis by LPL. In addition, we studied the influence of variation in the initial composition of both large, lipid-rich HDL and small, lipid-poor HDL on the outcome of the modelling.

In the first series of calculations, every kinetic parameter was either divided or multiplied by 2 and all the kinetics were calculated at fixed values of all other parameters. These calculations were performed at all four initial concentrations of HDL studied, including 5, 10, 20 and 40 mg/dL, and the results were plotted against HDL concentration. The appearance of the maximum of the accumulation of FC in HDL was found to be independent of the variation of 9 out of 13 kinetic parameters (Supplement Figure S1A,C,D,F–J,M,N). By contrast, two-fold increases in *V^max^_Tg_*, *k^LCAT^* and *k^coll^_FC_* as well as two-fold decrease in *k^grad^_FC_* abolished the maximum (Supplement Figure S1B,E,K,L). As the kinetic parameters of TG lipolysis by LPL are well-established, their variation may not meaningfully impact our model. On the other hand, our modelling showed that FC esterification by LCAT (described by *k^LCAT^*) as well as FC transfer between lipoproteins induced by a concentration gradient and during particle collision (described by *k^grad^_FC_* and *k^coll^_FC_*, respectively) all markedly contributed to FC accumulation in HDL upon VLDL lipolysis (Figure 5, Figure 6, Figure 7 and Figure 8).

Consistent with our earlier data [25], enhancing LCAT reaction reduced FC accumulation in HDL (Figure 8A). As the contribution of this process was particularly deleterious at low HDL concentrations and less so when HDL concentration was high (Figure 5A), the maximum at the dose-dependence of FC accumulation disappeared upon increasing *k^LCAT^* (Supplement Figure S1E). Contribution of collisional FC transfer to net FC accumulation in HDL increased with increasing HDL concentrations (Figure 5D). Consistent with this observation, increasing *k^coll^_FC_* preferentially enhanced FC accumulation at high HDL concentrations and abolished the maximum (Supplement Figure S1L). Finally, contribution of gradient-driven FC transfer was strongly decreased at high HDL concentrations, reflecting inversion of the concentration gradient between VLDL and HDL (Figure 5C). As a corollary, decreasing *k^grad^_FC_* particularly weakened this transfer, enhanced FC accumulation in HDL at high HDL concentrations and abolished the maximum at the dose-dependence of FC accumulation (Supplement Figure S1K). 

In the second series of calculations, initial concentration of each component of large, lipid-rich HDL was either decreased or increased to reflect their physiologically relevant variations, whereas total HDL concentration was kept constant (Supplement Table S1). These calculations were performed at all four initial concentrations of HDL studied, including 5, 10, 20 and 40 mg/dL. The appearance of the maximum of the accumulation of FC in HDL was found to be independent of the variation of the initial HDL content of CE, apoA-I and PL, whereas reduction of the initial content of FC abolished the maximum (Supplement Figure S2), suggesting that FC-poor HDL possess elevated capacity to accept exogenous FC.

In the last series of calculations, composition of each component of lipid-poor HDL was varied to reflect their variations reported in the literature [1] (Supplement Table S2). These calculations were equally performed at all four initial concentrations of HDL studied. The appearance of the maximum of the accumulation of FC in HDL was found to be independent of the variation of the initial content of FC, apoA-I and PL in small, lipid-poor HDL (Supplement Figure S3). Together, the results of our variational analysis showed that our conclusions were only weakly influenced by a limited set of parameters and that these effects were consistent with the rest of the data obtained.

## Figures and Tables

**Table 1:** Lipoprotein components included in the model.

Symbol	Description
Alp	lipid-poor apoA-I
A_α_	apoA-I in HDL_α_
CE_α_	CE in HDL_α_
FC_α_	FC in HDL_α_
Pl_α_	PL in HDL_α_
Tg_α_	TG in HDL_α_
TC_α_	TC in HDL_α_
A_V_	apoA-I in VLDL
CE_V_	CE in VLDL
FC_V_	FC in VLDL
Pl_V_	PL in VLDL
Tg_V_	TG in VLDL
Dg	diglyceride
Mg	monoglyceride
FFA	free fatty acid
Gl	glycerol

**Table 2:** Reactions included in the model and their rates.

No.	Reaction	Description	Reaction Rate
1	$Tg_{V}\Rightarrow Gl+FFA$	Decomposition of VLDL-TG by LPL during lipolysis	$\frac{V_{Tg}^{max}\cdot Tg_{V}\left(t\right)}{k_{Tg}^{m}+Tg_{V}\left(t\right)}$
2	$CE_{\alpha }\Rightarrow CE_{V}$	CE transfer from HDL to VLDL mediated by CETP	$k^{CETP}\left(t\right)\cdot CE_{\alpha }\left(t\right)$
3	$CE_{\alpha }\Rightarrow CE_{V}$	CE transfer from HDL to VLDL mediated by PLTP	$k_{CE}^{PLTP}\cdot CE_{\alpha }\left(t\right)$
4	$FC_{\alpha }\Rightarrow CE_{\alpha }$	Conversion of FC to CE in HDL by LCAT	$\frac{k^{LCAT}\cdot FC_{V}^{rem}\left(t\right)}{k_{FC}^{m}+FC_{V}^{rem}\left(t\right)}$
5	$FC_{V}\Rightarrow FC_{\alpha }$	Transfer of FC from VLDL to HDL under the action of concentration gradient	$k_{FC}^{grad}\left(t\right)\cdot FC_{V}\left(t\right)$
6	$Pl_{V}\Rightarrow Pl_{\alpha }$	Transfer of PL from VLDL to HDL under the action of concentration gradient	$k_{Pl}^{grad}\left(t\right)\cdot Pl_{V}\left(t\right)$
7	$A_{\alpha }\Rightarrow \emptyset$	Dissociation of apoA-I from HDL	$k^{diss}\left(t\right)\cdot A_{\alpha }\left(t\right)$
8	$FC_{V}\Rightarrow FC_{\alpha }$	FC transfer from VLDL to HDL during lipoprotein collision	$k_{FC}^{coll}\cdot FC_{\alpha }\left(t\right)\cdot FC_{V}\left(t\right)$
9	$A_{lp}\Rightarrow HDL_{\alpha }$	Fusion of Alp with HDL	$k^{fus}\left(t\right)\cdot A_{lp}\left(t\right)$
10	$Pl_{V}\Rightarrow Pl_{\alpha }$	PL transfer from VLDL to HDL during lipoprotein collision	$k_{Pl}^{coll}\cdot Pl_{\alpha }\left(t\right)\cdot Pl_{V}\left(t\right)$
11	$Pl_{V}\Rightarrow Pl_{\alpha }$	PL transfer from VLDL to HDL mediated by PLTP	$k_{Pl}^{PLTP}\cdot Pl_{V}\left(t\right)$
12	$A_{lp}\Rightarrow A_{lp}$	Transfer from VLDL to HDL of Alp released during lipolysis	$A_{lp}^{rem}\left(t\right)$
13	$A_{V}\Rightarrow A_{\alpha }$	Transfer from VLDL to HDL of apoA-I released during lipolysis	$A_{V}^{rem}\left(t\right)$

**Table 3:** Parameters used in the model.

Symbol	Description	Value
*m_Tg_*	Molar mass of TG	850 g/mol
*m_CE_*	Molar mass of CE	649 g/mol
*m_FC_*	Molar mass of FC	387 g/mol
*m_Pl_*	Molar mass of PL	775 g/mol
*m_A_*	Molar mass of apoA-I	28,500 g/mol
*m_Alp_*	Molar mass of lipid-poor apoA-I	69,180 g/mol
*S_V_*	Surface area of VLDL	9498 nm^2^
*S* *c_V_*	Surface area of VLDL core	8154 nm^2^

**Table 4:** Variables and rate constants used in the model.

Symbol	Description	Unit
$V_{Tg}^{max}$	Maximal reaction rate of TG lipolysis by LPL	$\frac{\left(mg/dL\right)}{h}$
$k_{Tg}^{m}$	$\mathrm{TG}\;\mathrm{concentration}\;\mathrm{at}\;V_{Tg}^{max}/2$	mg/dL
$k^{CETP}$	Rate constant of CE transfer from HDL to VLDL by CETP	1/h
$V_{FC}^{max}=k^{LCAT}$	Maximal reaction rate of FC conversion to CE by LCAT	$\frac{\left(mg/dL\right)}{h}$
$k_{FC}^{m}$	$\mathrm{FC}\;\mathrm{concentration}\;\mathrm{at}\;V_{FC}^{max}/2$	mg/dL
$k^{fus}$	Fusion rate constant	1/h
$k_{CE}^{PLTP}$	Rate constant of CE transfer from HDL to VLDL by PLTP	1/h
$k_{FC}^{grad}$	Rate constant of FC transfer from VLDL to HDL under the action of concentration gradient	1/h
$k_{FC}^{coll}$	Rate constant of FC transfer from VLDL to HDL upon their collision	$\frac{1}{\left(mg/dL\right)\cdot h}$
$k^{diss}$	Dissociation rate constant	1/h
$k_{Pl}^{grad}$	Rate constant of PL transfer from VLDL to HDL under the action of concentration gradient	1/h
$k_{Pl}^{coll}$	Rate constant of PL transfer from VLDL to HDL upon their collision	$\frac{1}{\left(mg/dL\right)\cdot h}$
$k_{Pl}^{PLTP}$	Rate constant of PL transfer from HDL to VLDL by PLTP	1/h

**Table 5:** Initial concentrations of lipoprotein components.

[HDL_α_], mg/dL	[Tg_V_], mg/dL	[CE_α_], mg/dL	[FC_α_], mg/dL	[Alp], mg/dL	[A_α_], mg/dL	[Pl_α_], mg/dL	[Tg_α_], mg/dL
5	30	0.8	0.2	0	2	1.25	0.225
10	30	1.6	0,4	0	4	2.5	0.45
20	30	3.2	0.8	0	8	5	0.9
40	30	6.4	1.6	0	16	10	1.8

**Table 6:** The values of the rate constants used in the model.

$V_{Tg}^{max},\left(mg/dL\right)/h$	12
$k_{Tg}^{m},mg/dL$	9.43
$k^{CETP},\;1/h$	0.020
$k^{LCAT},\left(mg/dL\right)/h$	0.46
$k_{FC}^{m},mg/dL$	0.00174
$k^{fus},\;1/h$	1.0
$k_{CE}^{PLTP},\;1/h$	0.002
$k_{FC}^{grad},\;1/h$	0.5
$k_{FC}^{coll},\frac{1}{\left(mg/dL\right)\cdot h}$	0.1
$k^{diss},\;1/h$	0.03
$k_{Pl}^{grad},\;1/h$	1.0
$k_{Pl}^{coll},\frac{1}{\left(mg/dL\right)\cdot h}$	0.03
$k_{Pl}^{PLTP},\;1/h$	0.2

## Data Availability

Data will be made available on reasonable request. The data are not publicly available due to privacy.

## Supplementary Materials

The following supporting information can be downloaded at: https://www.mdpi.com/article/10.3390/metabo12070623/s1, Figure S1: Influence of variations in kinetic parameters on the dose-dependence of FC accumulation in HDL during VLDL lipolysis by LPL in the presence of HDL., Figure S2: Influence of variations in the initial composition of large, lipid-rich HDL on the dose-dependence of FC accumulation in HDL during VLDL lipolysis by LPL in the presence of HDL., Figure S3: Influence of variations in the initial composition of small, lipid-poor HDL on the dose-dependence of FC accumulation in HDL during VLDL lipolysis by LPL in the presence of HDL; Table S1: Variations in the initial composition (mg/dL) of large, lipid-rich HDL., Table S2: Variations in the initial composition (mol/mol HDL) of small, lipid-poor HDL. Refs [1,25,36,51,52,53,54,55] are cited in Supplementary Materials.
