# Supplementary material for: Mathematical Modelling of Material Transfer to High-Density Lipoprotein (HDL) upon Triglyceride Lipolysis by Lipoprotein Lipase: Relevance to Cardioprotective Role of HDL
Source: Metabolites. 2022 Jul 6;12(7):623. doi: 10.3390/metabo12070623 (PMC9317498; doi:10.3390/metabo12070623)
Supplement: Supplementary file 1 [file metabolites-12-00623-s001.zip › metabolites-1490151-supplementary.pdf]

Supplement Table S1. Variations in the initial composition (mg/dl) of large, lipid-rich HDL.

HDL concentration, 5 mg/dl

	Standard composition	Reduced CE	Elevated CE	Reduced FC	Elevated FC	Reduced apoA-I	Elevated apoA-I	Reduced PL	Elevated PL
CE	0.8	0.7	0.9	0.8	0.8	0.85	0.75	0.8	0.8
FC	0.2	0.2	0.2	0.1	0.3	0.2	0.2	0.2	0.2
ApoA-I	2.0	2.05	1.95	2.05	1.95	1.9	2.1	2.1	1.9
PL	1.25	1.30	1.20	1.30	1.20	1.30	1.20	1.15	1.35

HDL concentration, 10 mg/dl

	Standard composition	Reduced CE	Elevated CE	Reduced FC	Elevated FC	Reduced apoA-I	Elevated apoA-I	Reduced PL	Elevated PL
CE	1.6	1.4	1.8	1.65	1.55	1.7	1.5	1.6	1.6
FC	0.4	0.4	0.4	0.2	0.6	0.4	0.4	0.4	0.4
ApoA-I	4.0	4.1	3.9	4.1	3.9	3.8	4.2	4.2	3.8
PL	2.5	2.6	2.4	2.55	2.45	2.6	2.4	2.3	2.7

HDL concentration, 20 mg/dl

	Standard composition	Reduced CE	Elevated CE	Reduced FC	Elevated FC	Reduced apoA-I	Elevated apoA-I	Reduced PL	Elevated PL
CE	3.2	2.8	3.6	3.3	3.1	3.4	3.0	3.2	3.2
FC	0.8	0.8	0.8	0.4	1.2	0.8	0.8	0.8	0.8
ApoA-I	8.0	8.2	7.8	8.2	7.8	7.6	8.4	8.4	7.6
PL	5.0	5.2	4.8	5.1	4.9	5.2	4.8	4.6	5.4

HDL concentration, 40 mg/dl

	Standard composition	Reduced CE	Elevated CE	Reduced FC	Elevated FC	Reduced apoA-I	Elevated apoA-I	Reduced PL	Elevated PL
CE	6.4	5.6	7.2	6.6	6.2	6.8	6.0	6.4	6.4
FC	1.6	1.6	1.6	0.8	2.4	1.6	1.6	1.6	1.6
ApoA-I	16.0	16.4	15.6	16.4	15.6	15.2	16.8	16.8	15.2
PL	10.0	10.4	9.6	10.2	9.8	10.4	9.6	9.2	10.8

Physiologically relevant variations in HDL composition similar to those observed in patients with cardiometabolic diseases [1-6] were studied at a constant total concentration of HDL. As lipid accumulation in HDL was not influenced by the initial TG content of large HDL particles, variations in this parameter were not studied.

Supplement Table S2. Variations in the initial composition (mol/mol HDL) of small, lipid-poor HDL.

	Standard composition	Reduced FC	Elevated FC	Reduced PL	Elevated PL	Elevated apoA-I
FC	5	2.5	10	5	5	5
PL	50	50	50	25	100	50
ApoA-I	1	1	1	1	1	2

Physiologically relevant variations in HDL composition similar to those reported for plasma pre-beta HDL [7] were studied.

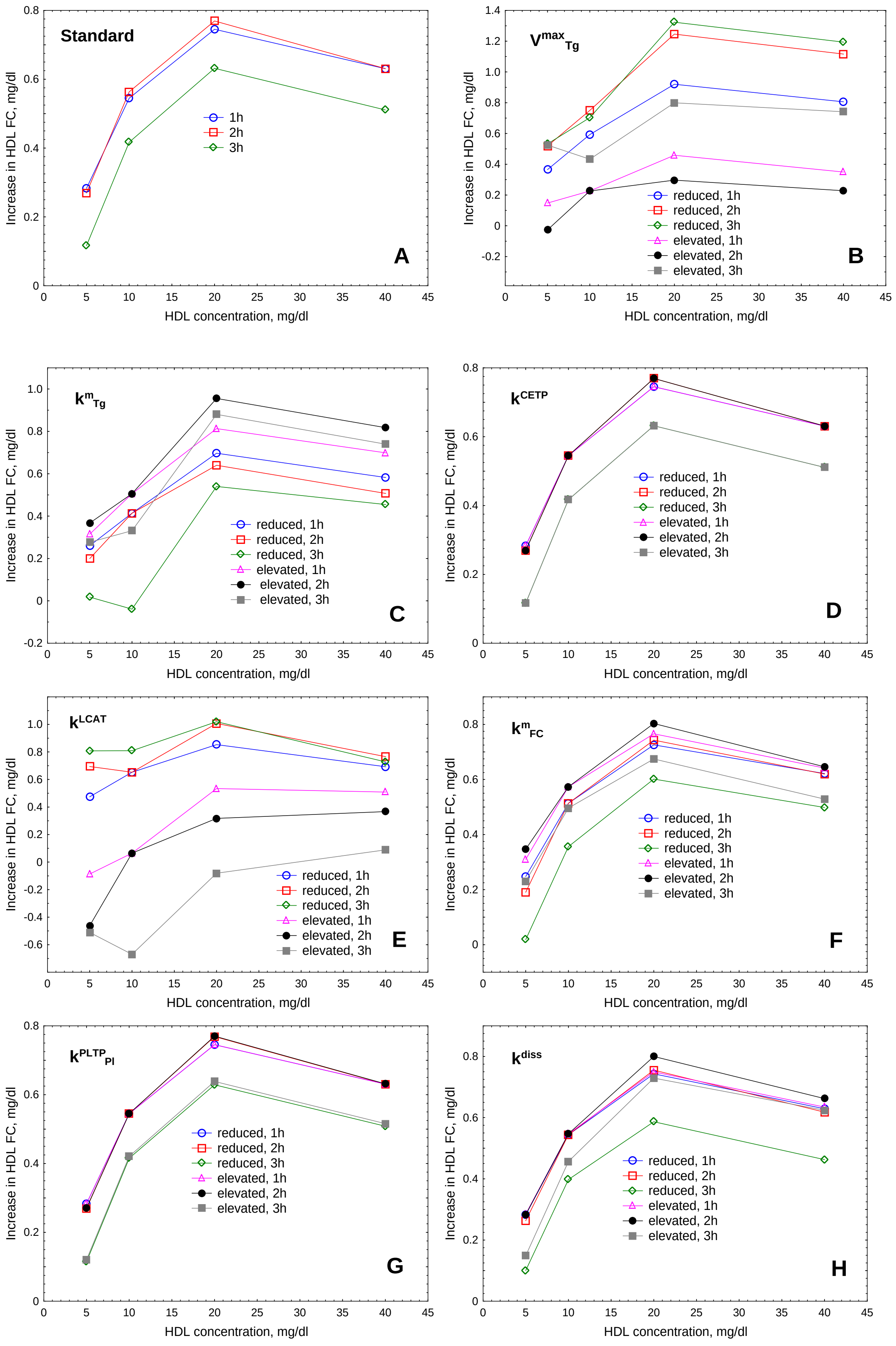
Standard
1h
2h
3h
A
V^{max}_{Tg}
B
k^{m}_{Tg}
C
k^{CETP}
D
k^{LCAT}
E
k^{m}_{FC}
F
k^{PLTP}_{Pl}
G
k^{diss}
H
reduced, 1h
reduced, 2h
reduced, 3h
elevated, 1h
elevated, 2h
elevated, 3h
Increase in HDL FC, mg/dl
HDL concentration, mg/dl

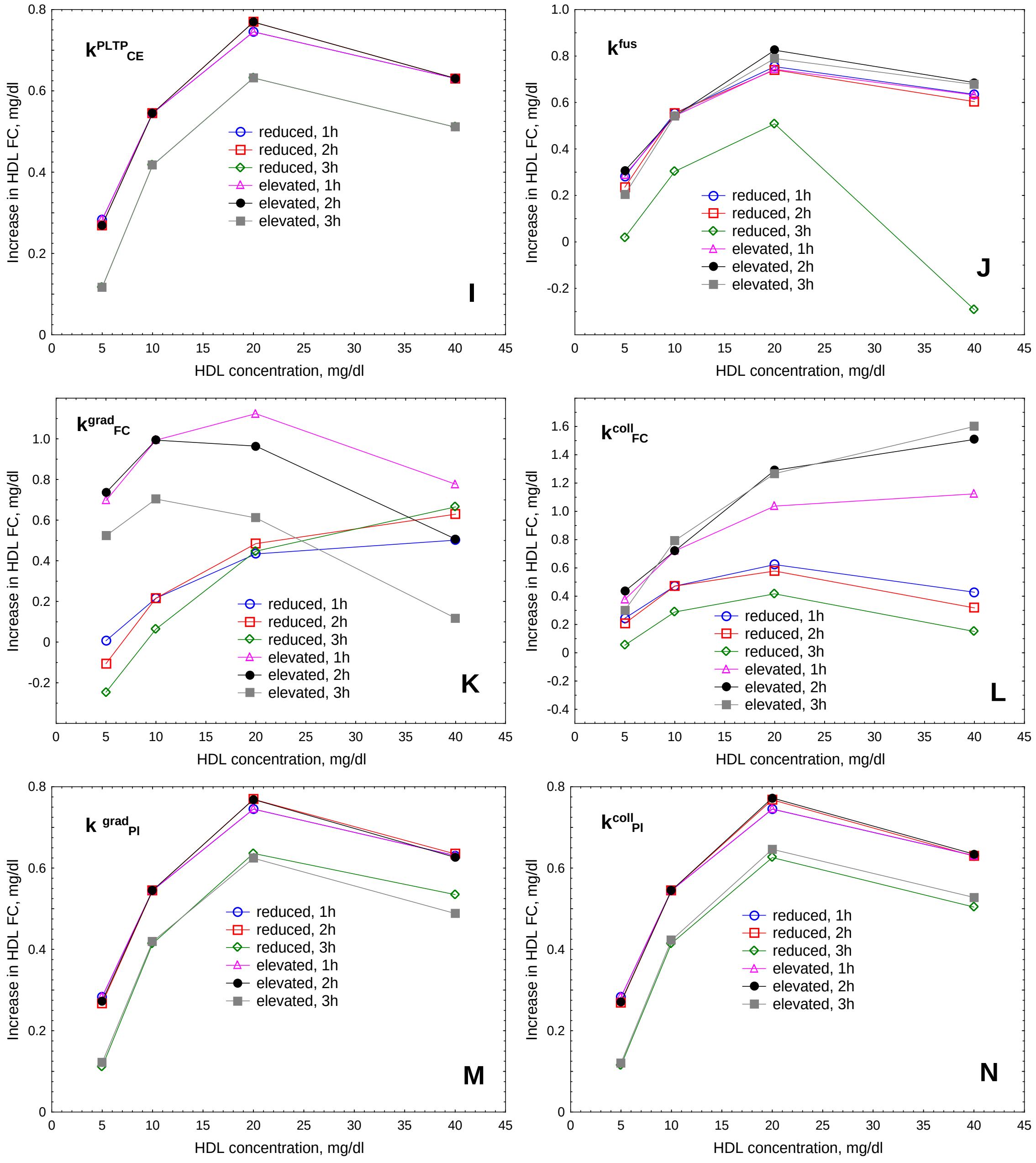

Supplement Figure S1. Influence of variations in kinetic parameters on the dose-dependence of FC accumulation in HDL during VLDL lipolysis by LPL in the presence of HDL. Mathematical modelling was performed under conditions of in vitro TGRL lipolysis described elsewhere [8] for the reaction time of 1 (blue circles or pink triangles), 2 (red squares or black circles) and 3 (green diamonds or grey squares) h. Every kinetic parameter (shown in the upper left corner of each panel) was either divided or multiplied by 2 and FC kinetics were calculated at fixed values of all other parameters. In order to calculate FC

accumulation in HDL, FC content at baseline (t=0) was subtracted from the results of the modelling. Initial concentrations: HDL, 5, 10, 20 and 40 mg total mass/dl; VLDL-TG, 30 mg/dl; LPL, 190 U/ml.

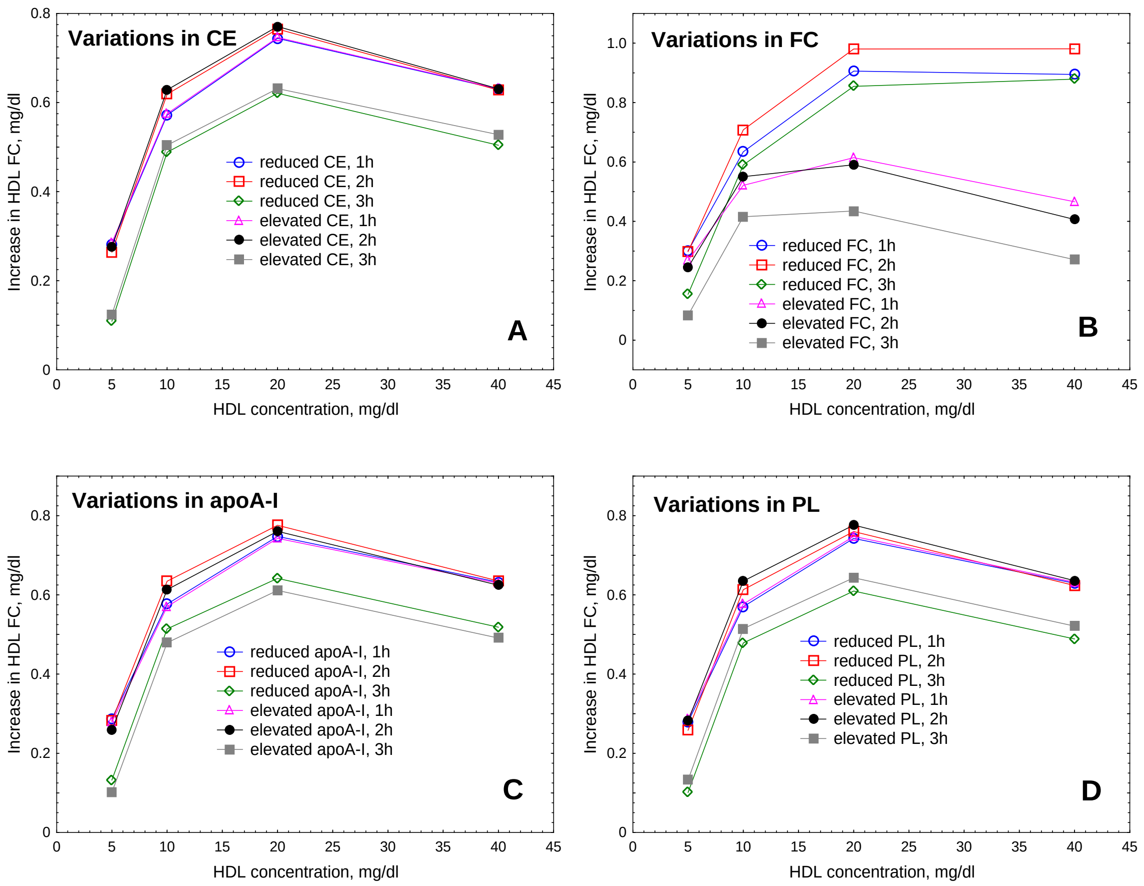

Supplement Figure S2. Influence of variations in the initial composition of large, lipid-rich HDL on the dose-dependence of FC accumulation in HDL during VLDL lipolysis by LPL in the presence of HDL. Mathematical modelling was performed under conditions of in vitro TGRL lipolysis described elsewhere [8] for the reaction time of 1 (blue circles or pink triangles), 2 (red squares or black circles) and 3 (green diamonds or grey squares) h. Initial concentration of each component of large, lipid-rich HDL was either decreased or increased to reflect their physiologically relevant variations [1-6], while total HDL concentration was kept constant (Supplement Table S1) and FC kinetics were calculated at fixed values of all other parameters. In order to calculate FC accumulation in HDL, FC content at baseline (t=0) was subtracted from the results of the modelling. Initial concentrations: HDL, 5, 10, 20 and 40 mg total mass/dl; VLDL-TG, 30 mg/dl; LPL, 190 U/ml.

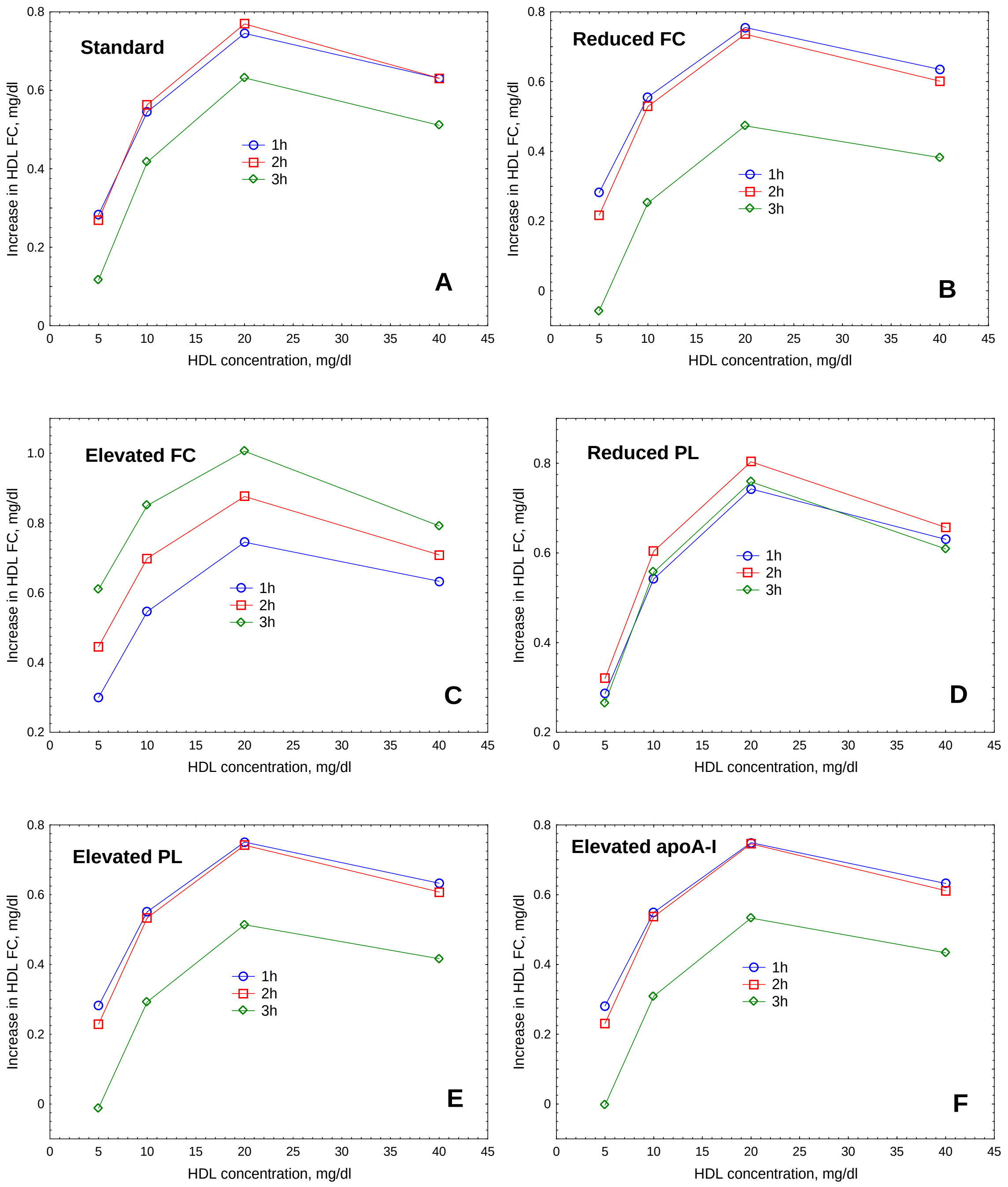

Supplement Figure S3. Influence of variations in the initial composition of small, lipid-poor HDL on the dose-dependence of FC accumulation in HDL during VLDL lipolysis by LPL in the presence of HDL. Mathematical modelling was performed under conditions of in vitro TGRL lipolysis described elsewhere [8] for the reaction time of 1 (blue circles), 2 (red squares) and 3 (green diamonds) h. Composition of each component of lipid-poor HDL was varied to reflect their variations reported in the literature [7] (Supplement Table S2) and

FC kinetics were calculated at fixed values of all other parameters. In order to calculate FC accumulation in HDL, FC content at baseline (t=0) was subtracted from the results of the modelling. Initial concentrations: HDL, 5, 10, 20 and 40 mg total mass/dl; VLDL-TG, 30 mg/dl; LPL, 190 U/ml.

References

1. Kontush, A.; Chantepie, S.; Chapman, M.J. Small, dense HDL particles exert potent protection of atherogenic LDL against oxidative stress. *Arterioscler Thromb Vasc Biol* **2003** *23*, 1881-8.
2. Hansel, B.; Giral, P.; Nobecourt, E.; Chantepie, S.; Bruckert, E.; Chapman, M.J.; Kontush, A. Metabolic syndrome is associated with elevated oxidative stress and dysfunctional dense high-density lipoprotein particles displaying impaired antioxidative activity. *J Clin Endocrinol Metab* **2004** *89*, 4963-71.
3. Kontush, A.; de Faria, E.C.; Chantepie, S.; Chapman, M.J. A normotriglyceridemic, low HDL-cholesterol phenotype is characterised by elevated oxidative stress and HDL particles with attenuated antioxidative activity. *Atherosclerosis* **2005** *182*, 277-85.
4. Nobecourt, E.; Jacqueminet, S.; Hansel, B.; Chantepie, S.; Grimaldi, A.; Chapman, M.J.; Kontush, A. Defective antioxidative activity of small, dense HDL particles in type 2 diabetes: Relationship to elevated oxidative stress and hyperglycemia. *Diabetologia* **2005** *48*, 529-38.
5. de Souza, J.A.; Vindis, C.; Hansel, B.; Negre-Salvayre, A.; Therond, P.; Serrano Jr, C.V.; Chantepie, S.; Salvayre, R.; Bruckert, E.; Chapman, M.J.; Kontush, A. Metabolic syndrome features small, apolipoprotein A-I-poor, triglyceride-rich HDL3 particles with defective anti-apoptotic activity. *Atherosclerosis* **2008** *197*, 84.
6. Chantepie, S.; Bochem, A.E.; Chapman, M.J.; Hovingh, G.K.; Kontush, A. High-density lipoprotein (HDL) particle subpopulations in heterozygous cholesteryl ester transfer protein (CETP) deficiency: maintenance of antioxidative activity. *PLoS One* **2012** *7*, e49336.
7. Kontush, A.; Chapman, M.J. *High-Density Lipoproteins: Structure, Metabolism, Function and Therapeutics*. Wiley & Sons; New York, 2012; 648 p.
8. Feng, M.; Darabi, M.; Tubeuf, E.; Canicio, A.; Lhomme, M.; Frisdal, E.; Lanfranchi-Lebreton, S.; Matheron, L.; Rached, F.; Ponnaiah, M.; Serrano, C.V., Jr.; Santos, R.D.; Brites, F.; Bolbach, G.; Gautier, E.; Huby, T.; Carrie, A.; Bruckert, E.; Guerin, M.; Couvert, P.; Giral, P.; Lesnik, P.; Le Goff, W.; Guillas, I.; Kontush, A. Free cholesterol transfer to high-density lipoprotein (HDL) upon triglyceride lipolysis underlies the U-shape relationship between HDL-cholesterol and cardiovascular disease. *European journal of preventive cardiology* **2020** *27*, 1606-16.
